# Exploring the heterogeneity of effects of corticosteroids on acute respiratory distress syndrome: a systematic review and meta-analysis

**DOI:** 10.1186/cc13819

**Published:** 2014-04-07

**Authors:** Sheng-Yuan Ruan, Hsien-Ho Lin, Chun-Ta Huang, Ping-Hung Kuo, Huey-Dong Wu, Chong-Jen Yu

**Affiliations:** 1 Graduate Institute of Epidemiology and Preventive Medicine, National Taiwan University, No.17 Xu-Zhou Road, Taipei 10020, Taiwan; 2 Division of Pulmonary and Critical Care Medicine, Department of Internal Medicine, National Taiwan University Hospital, Taipei, Taiwan

## Abstract

**Introduction:**

The effectiveness of corticosteroid therapy on the mortality of acute respiratory distress syndrome (ARDS) remains under debate. We aimed to explore the grounds for the inconsistent results in previous studies and update the evidence.

**Methods:**

We searched MEDLINE, Cochrane Central Register of Controlled Trials and Web of Science up to December 2013. Eligible studies included randomized clinical trials (RCTs) and cohort studies that reported mortality and that had corticosteroid nonusers for comparison. The effect of corticosteroids on ARDS mortality was assessed by relative risk (RR) and risk difference (RD) for ICU, hospital, and 60-day mortality using a random-effects model.

**Results:**

Eight RCTs and 10 cohort studies were included for analysis. In RCTs, corticosteroids had a possible but statistically insignificant effect on ICU mortality (RD, −0.28; 95% confidence interval (CI), −0.53 to −0.03 and RR, 0.55; 95% CI, 0.24 to 1.25) but no effect on 60-day mortality (RD, −0.01; 95% CI, −0.12 to 0.10 and RR, 0.97; 95% CI, 0.75 to 1.26). In cohort studies, corticosteroids had no effect on ICU mortality (RR, 1.05; 95% CI, 0.74 to 1.49) but non-significantly increased 60-day mortality (RR, 1.30; 95% CI, 0.96 to 1.78). In the subgroup analysis by ARDS etiology, corticosteroids significantly increased mortality in influenza-related ARDS (three cohort studies, RR, 2.45, 95% CI, 1.40 to 4.27).

**Conclusions:**

The effects of corticosteroids on the mortality of ARDS differed by duration of outcome measures and etiologies. Corticosteroids did not improve longer-term outcomes and may cause harm in certain subgroups. Current data do not support routine use of corticosteroids in ARDS. More clinical trials are needed to specify the favorable and unfavorable subgroups for corticosteroid therapy.

## Introduction

Despite advances in critical care medicine over the past decades, the mortality rate for acute respiratory distress syndrome (ARDS) remains high [1-3]. Because dysregulated inflammation is the cardinal feature of ARDS [2,4], systemic corticosteroids have been considered a potentially beneficial therapy. However, previous randomized trials have failed to provide convincing evidence to prove the efficacy of corticosteroids in decreasing the mortality of ARDS [5-8]. Only secondary outcomes, such as oxygenation improvement and reduction of the duration of mechanical ventilation, have shown consistent findings in favor of corticosteroid therapy.

Published meta-analyses about corticosteroid therapy for ARDS reported inconsistent conclusions [9-13]. Different study selections and heterogeneity on mortality endpoints and etiologies of ARDS may account for the inconsistent study results in previous meta-analyses. Measuring the treatment effects at short-term or longer-term follow-up may influence study results, because therapeutic effects of corticosteroids develop early but some adverse effects, such as infection, develop late. Using short-term outcome as the study endpoint may underestimate the risk of corticosteroid therapy and overestimate the overall benefit. In addition, the mechanisms of lung injury and fibroproliferative response to injury vary in pulmonary and extrapulmonary ARDS [14]. Therefore, the treatment response to corticosteroids in ARDS may be different in ARDS of different etiologies. However, the influence of the etiologies of ARDS on outcomes of corticosteroid therapy has not been evaluated in previous studies.

We conducted a systematic review and meta-analysis of corticosteroid therapy in ARDS with the aim of updating the best available evidence and exploring the source of observed heterogeneity.

## Materials and Methods

### Search strategy

This systematic review was conducted using an *a priori* published protocol submitted to the PROSPERO website (Registration No.: CRD42012002583) and reported according to the Preferred Reporting Items for Systematic Reviews and Meta-Analyses (PRISMA) criteria [15]. No institutional review board (IRB) approval or consents were needed for this systematic review because it evaluated published studies. We searched MEDLINE via the NCBI Entrez system, Cochrane Central Register of Controlled Trials (CENTRAL) and Web of Science (WOS) up to December 2013. We also screened the bibliographies of retrieved studies and recent review articles to identify additional trials.

Keyword search was performed in MEDLINE, CENTRAL and WOS using the following terms: ‘corticosteroids’ AND (‘ALI’ OR ‘acute lung injury’ OR ‘ARDS’ OR ‘acute respiratory distress syndrome’). The search was then limited to human studies. We also used MeSH term search in MEDLINE with the following terms: (‘respiratory distress syndrome, adult’ OR ‘acute lung injury’) AND (‘hydroxycorticosteroids’ OR ‘glucocorticoids’). No language restrictions were applied.

The inclusion criteria included randomized controlled trial (RCT) and cohort study designs that reported mortality outcomes and had corticosteroid nonusers for comparison.

### Quality assessment and data extraction

Two investigators (SYR and CTH) independently extracted data from the included studies into standardized data recording forms. Quality assessment of these studies was done using the Cochrane Risk of Bias Tool for RCTs and the Newcastle-Ottawa Quality Assessment Scale for cohort studies [16,17].

### Outcome measurement

The primary study endpoint was all-cause mortality. Different mortality measures were reported in the studies, including ICU mortality, hospital mortality and 60-day mortality. According to the duration of mean ICU and hospital stay reported in the included studies [7,8,18], we classified ICU mortality as short-term outcome, hospital mortality as a mid-term outcome, and 60-day mortality as a longer-term outcome. Additionally, two studies reported 14-day and 45-day mortality [5,19], and these two outcome measures were classified as ICU mortality and 60-day mortality, respectively. The secondary study endpoint was nosocomial infections related to corticosteroid therapy.

### Statistical analysis

Relative risk (RR) and risk difference (RD) were used as measurements of association. Outcome measures were pooled using a random-effects model because of anticipated heterogeneity among included studies. We treated risk ratio and hazard ratio as RR when pooling across studies. We estimated the point estimate and 95% confidence interval (95% CI) of the summary effect estimate (RR or RD). RCTs and cohort studies were analyzed separately. When one arm of a study contained no events, 0.5 was added to all cells of the two-by-two table. Heterogeneity was explored using the Q statistic and I^2^. Heterogeneity was considered low, moderate, and high by I^2^ values of 25%, 50%, and 75%, respectively [20]. We hypothesized that the treatment response to corticosteroids in ARDS patients may vary by different mortality endpoints, by different etiologies of ARDS, and by different timing for starting treatment. We, therefore, conducted subgroup analyses to explore whether the treatment response varied by these variables of interest. All tests were two-sided and *P* values of <0.05 were deemed significant. The data were analyzed using Stata software, V.11 (StataCorp).

## Results

**Figure 1:**
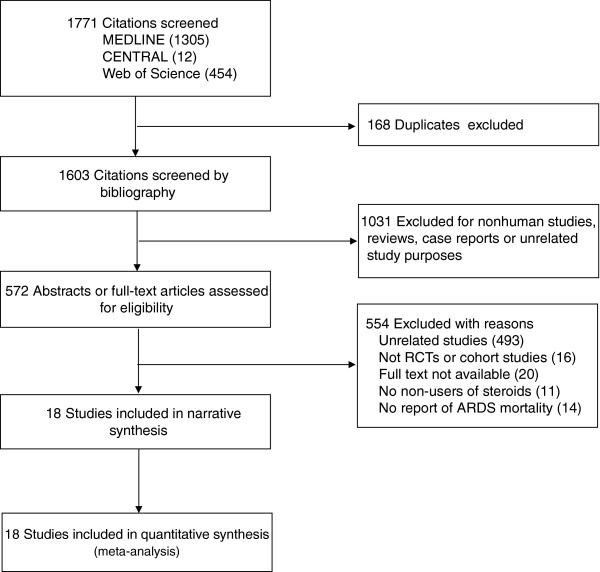
Number of studies evaluated at each stage of the systematic review.

We identified 1,771 citations from the search of electronic databases. Using the predefined inclusion and exclusion criteria (Figure 1), seven RCTs [5-8,19,21,22], one *post-hoc* analysis of RCT [23], and 10 cohort studies were included for evaluation [18,24-32]. The *post-hoc* analysis was classified as RCT for the analysis. Of the eight RCTs, three trials studied preventive rather than therapeutic use of corticosteroids in ARDS. A total of 1,474 subjects were analyzed, including 725 from RCTs and 749 from cohort studies. The details of the included studies are summarized in Additional file 1: e-Table S1. Quality assessment of the included studies suggested a low risk of bias in most RCTs (Additional file 1: e-Table S2) but the comparability and representativeness of the study patients were concerns in cohort studies (Additional file 1: e-Table S3, e-Table S4).

### Mortality outcomes

**Figure 2:**
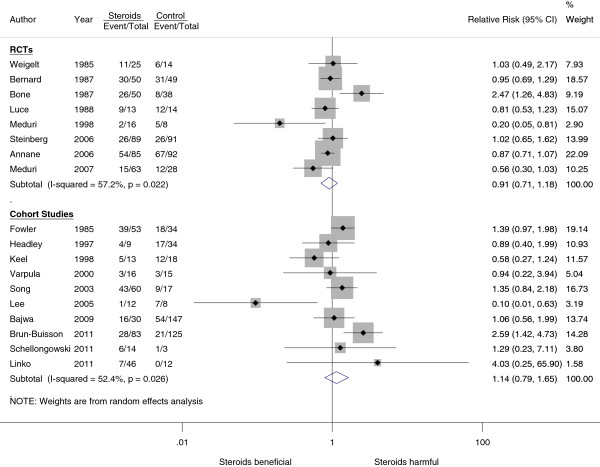
Effect of corticosteroids on hospital or 60-day mortality.

Figure 2 shows the effects of corticosteroid therapy on mortality. Pooled results from all RCTs suggested that corticosteroids had no significant effect on mortality (RR, 0.91; 95% C.I., 0.71 to 1.18). Visual inspection of the forest plot and the estimated I^2^ (57%) revealed moderate heterogeneity among the included studies. To explore the influence of different mortality endpoints, we conducted subgroup analysis by different mortality endpoints (Table 1). The results suggested that corticosteroids had a possible but statistically insignificant effect on short-term (ICU) mortality (RR, 0.55; 95% CI, 0.24 to 1.25 and RD, −0.28; 95% CI, −0.53 to −0.03) but did not decrease 60-day mortality. Within-study observations in those studies reporting two mortality outcomes by different follow-up durations also suggested similar findings, that the disadvantage of corticosteroid therapy increased by prolonging the follow-up period (Additional file 1: e-Table S5). In cohort studies, corticosteroids had no effect on ICU mortality but there was a trend of increased mortality on 60-day mortality (Table 1). A funnel plot of standard error versus risk ratio for mortality did not suggest significant publication bias (Additional file 2: e-Figure S1, e-Figure S2).

**Table 1:** Short-term and longer-term effects of corticosteroids on mortality

**Mortality endpoints**	**Number of**		**Relative risk**		**Risk difference**	
	**Studies**	**Patients**	**Effect size (95% CI)**	**I**^**2**^	**Effect size (95% CI)**	**I**^**2**^
**Randomized controlled trials**^**a**^						
ICU mortality	3	292	0.55 (0.24 to 1.25)	75%	−0.28 (−0.53 to −0.03)*	76%
Hospital mortality	2	201	0.49 (0.12 to 2.07)	77%	−0.26 (−0.65 to 0.13)	75%
60-day mortality	2	279	0.97 (0.75 to 1.26)	0%	−0.01 (−0.12 to 0.10)	0%
**Cohort studies**						
ICU mortality	5	226	1.05 (0.74 to 1.49)	1%	Not calculable	-
Hospital mortality	4	317	1.00 (0.23 to 4.34)	75%	Not calculable	-
60-day mortality	2	264	1.30 (0.96 to 1.78)	0%	Not calculable	-

^**a**^Trials testing preventive therapy of corticosteroids for acute respiratory distress syndrome (ARDS) were excluded. Some studies provided data on both short-term and longer-term mortality. For cohort studies, only pooled relative risk was provided because adjusted results were needed for data pooling *P-value <0.05.

### Etiologies of ARDS

We also conducted a subgroup analysis by different etiologies of ARDS. For this analysis, we classified the study population into four groups: unselected ARDS, sepsis-related ARDS (infection-related ARDS >70% of cases), influenza-related ARDS, and post-operative ARDS. Table 2 summarizes the treatment outcome of corticosteroids in these four groups of ARDS patients. The responses to corticosteroid therapy differed among different etiologies of ARDS. Notably, corticosteroids significantly increased mortality in influenza-related ARDS (RR, 2.44; 95% CI, 1.4 to 4.27). One cohort study reported good treatment outcome in patients with post-operative ARDS but the sample size was very small [29].

**Table 2:** Effects of corticosteroids on mortality in different etiologies of ARDS

**Etiology of ARDS**^**a**^	**Number of studies**	**Number of patients**	**Relative risk (95% CI)**	**I**^**2**^
**Unselected ARDS**				
Randomized controlled trials	3	370	0.88 (0.65 to 1.18)	28%
Cohort studies	4	238	1.12 (0.78 to 1.60)	40%
**Influenza-related ARDS**^**b**^				
Cohort studies	3	283	2.45 (1.40 to 4.27) **	0%
**Sepsis-related ARDS**				
Randomized controlled trials	2	201	0.50 (0.12 to 2.02)	76%
Cohort studies	2	208	1.04 (0.58 to 1.85)	0%
**Post-operative ARDS**^**b**^				
Cohort studies	1	20	0.10 (0.01 to 0.63) *	-

^**a**^Excludes the studies of preventive use of corticosteroids; ^b^no randomized controlled trials available. *P-value <0.05. ** *P*-value <0.005. ARDS, acute respiratory distress syndrome.

### Infection risk of corticosteroids

**Figure 3:**
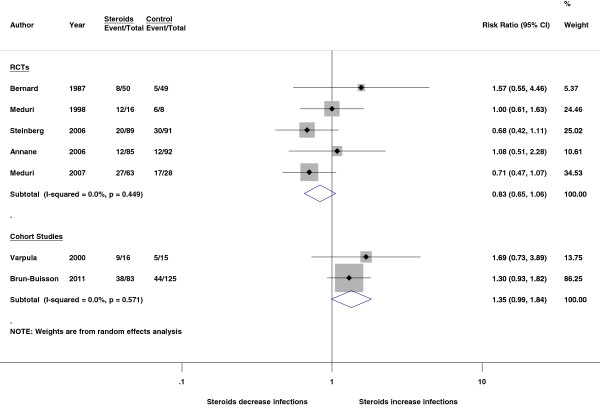
Effect of corticosteroids on infectious complications.

The definitions and observational durations of infectious complications varied greatly among included studies (Additional file 1: e-Table S6). The observed durations of infectious complications ranged from 7 days to 28 days. Pooled data showed conflicting findings that corticosteroids tend to increase infection risk in cohort studies (risk ratio, 1.35, 95% CI, 0.99 to 1.84) but decrease infection risk in RCTs (risk ratio, 0.83, 95% CI, 0.65 to 1.06) (Figure 3).

## Discussion

Our study found that the mortality outcomes of corticosteroid therapy in ARDS differed by duration of outcome measures. Corticosteroids had a possible but statistically insignificant effect on short-term mortality in RCTs but did not decrease longer-term mortality in either RCTs or cohort studies (Table 1). Within-study observation in studies reporting two mortality endpoints also suggested that the benefit of corticosteroid therapy decreased when follow-up was prolonged [7,8,23]. This raises a concern that corticosteroid therapy in ARDS may bring initial benefits by suppressing the inflammatory process and reducing alveolocapillary permeability [4,33,34], but the beneficial effects are soon counteracted by the delayed onset of adverse effects, such as immunosuppression and altered tissue repair [35,36]. We also found that the effect of corticosteroid therapy differed among different populations of ARDS patients. Corticosteroids may cause harm in certain ARDS subgroups, such as influenza-related ARDS. Taken together, current data do not support routine use of corticosteroids in ARDS. Given the heterogeneous nature of ARDS and the pleiotropic effects of corticosteroids, more clinical trials are needed to specify the favorable and unfavorable subgroups for corticosteroid therapy. For more comprehensive assessment of the effects of corticosteroid therapy, future studies should evaluate a mortality endpoint of adequate duration and the 60-day mortality used by the ARDSnet appears to be a reasonable study endpoint [6].

Our study demonstrated the diverse treatment effects of corticosteroids among different etiologies of ARDS (Table 2). It is biologically plausible that different etiologies of ARDS have different responses to corticosteroid therapy because the pulmonary fibroproliferative response to injury may occur in an injury-specific rather than a stereotyped manner [14]. The main damage targets differ in ARDS caused by different etiologies [37,38]. Therefore, it is not surprising that the efficacy of corticosteroid therapy differs among different etiologies. Additionally, our analysis showed that corticosteroids significantly increased mortality in influenza-related ARDS. The poorer outcome may be attributed to prolonged viral shedding and an increased risk of superinfection [39,40]. An expert review also advised against use of corticosteroids in the management of H1N1 influenza A infection [41].

The dosage and timing of corticosteroid therapy in ARDS has changed over the last decades. Based on the equivalent doses of methylprednisolone, studies before 1990 usually used a high daily dose (30 mg/kg) and short period (≤2 days) regimen to prevent or treat ARDS. In the following two decades, most studies used a protocol of daily dose of ≤2 mg/kg with a gradual taper. Some investigators suggested different treatment dosages for early ARDS and persistent ARDS in which a duration of ARDS ≤3 days was considered as early ARDS and ≥5 days as persistent or unresolving ARDS [4]. The treatment dose was suggested to be ≤1 mg/Kg for early ARDS [7,23], and 2 mg/Kg for persistent ARDS [6,8]. We summarized the treatment outcomes of corticosteroid therapy initiated at different stages of ARDS (Table 3). Our analysis found that patients with persistent ARDS seemed more likely to benefit from corticosteroid therapy. In addition, the subgroup analysis of the ARDSnet steroid study suggested against starting corticosteroid therapy >14 days after the onset of ARDS [6]. These findings suggest a subgroup of ARDS might benefit most from corticosteroid therapy: persistent ARDS with the onset of ARDS <14 days. Persistent ARDS indicates a specific subgroup or phenotype of ARDS that is characterized by exaggerated or unresolving lung inflammation [4]. In this subgroup, the benefit of corticosteroid therapy may outweigh the treatment risk. Dosage and administration schedules may also affect treatment outcomes of corticosteroids. However, it was difficult to evaluate the effects of these two factors in the study-level analysis. Cohort studies did not use standardized treatment protocols and individual-level data are needed to conduct such analyses. In RCTs, therapeutic trials usually used a low-dose regimen (1 to 2 mg/kg/day) and similar administration schedules.

**Table 3:** Subgroup analysis by timing of starting corticosteroid therapy

**Timing of starting steroids**	**Number of studies**	**Number of patients**	**Relative risk (95% CI)**	**I**^**2**^
**Preventive therapy**				
Randomized controlled trials	3	154	1.24 (0.57 to 2.72)	80%
**Early ARDS (≤3 days)**				
Randomized controlled trials	3	367	0.86 (0.71 to 1.04)	17%
Cohort studies	4	303	1.00 (0.24 to 4.20)	70%
**Persistent ARDS (≥5 days)**				
Randomized controlled trials	2	204	0.52 (0.11 to 2.52)	79%
Cohort studies	3	105	0.73 (0.44 to 1.23)	0%

Three studies were excluded in this analysis due to no report of treatment timing.

Published meta-analyses reached inconsistent conclusions on the role of corticosteroids for ARDS [9-13]. We summarize the study conclusions and study selection strategy of previous meta-analyses in Table 4. Among the included studies in these meta-analyses, an extremely protective effect for corticosteroids was observed in the two studies published by Meduri [7,8], but the effects were neutral and modest in other trials. In addition, inclusion or exclusion of trials of using corticosteroids in severe pneumonia into analysis also had an impact on the results of meta-analysis. Confalonieri and Meduri reported remarkable mortality reduction by corticosteroid therapy in a severe pneumonia study [42]. Previous meta-analyses reporting significant mortality reduction of corticosteroid therapy usually included this pneumonia study [12,13]. However, a large cohort study using a registry database reported that low-dose corticosteroids were associated with an increased mortality in pneumonia with septic shock [43].

**Table 4:** Comparisons of published meta-analyses

**Study (Ref.)**	**Included studies**	**Summary of study conclusion**	**Additional remarks**
Adhikari *et al*.(2004) [9]	Three RCTs	Early high-dose corticosteroids had no effect on early mortality. Corticosteroids given for late phase ARDS reduced hospital mortality.	Study interest not focused on corticosteroids; few studies and small sample size.
Agarwal *et al*. (2007) [10]	Four RCTs and two cohort studies	Current evidence does not support a role for corticosteroids in the management of ARDS in either the early or late stages of the disease.	Excluding the RCTs of preventive use of corticosteroids; including high-dose corticosteroid study.
Peter *et al*. (2008) [11]	Nine RCTs (eight RCTs for mortality analysis)	A definitive role of corticosteroids in the treatment of ARDS in adults is not established.	Including the RCTs of preventive use of corticosteroids; excluding pneumonia studies; using Bayesian random effects models for data pooling.
Tang *et al*. (2009) [12]	Four RCTs (three ARDS studies and one pneumonia study) and five cohort studies	The use of low-dose corticosteroids was associated with improved mortality and morbidity outcomes without increased adverse reactions.	Including a RCT of pneumonia; excluding studies of high-dose and preventive use of corticosteroids.
Lamontagne *et al*. (2010) [13]	Twelve RCTs (six ARDS studies and six pneumonia studies)	Corticosteroids administered within 14 days of disease onset may reduce all-cause mortality.	Including six studies of pneumonia.

ARDS, acute respiratory distress syndrome; RCTs, randomized controlled trials.

Systemic corticosteroid therapy may bring several unfavorable side effects [36,44], and one major concern in patients with ARDS is an increased risk of nosocomial infection secondary to immunosuppression. Because symptoms and signs of early infection may be masked by corticosteroids, previous RCTs performed intensive infection surveillance procedures during the study to reduce the risk of superinfection [6,7]. However, these intensive surveillance procedures are not always performed outside of clinical studies. Our analysis showed that the infection risks reported in RCTs and cohort studies were conflicting (Figure 3). It is not known whether restrictive patient selection and infection surveillance procedures in RCTs played a role in making such a difference. Similar to the concern for mortality outcomes, the infection risk of corticosteroid therapy should be evaluated in an adequate time frame because the immunosuppressive effect may develop late in the clinical course. However, most studies evaluated infectious complications in a short duration (Additional file 1: e-Table S6) and the infection risk of corticosteroid therapy might, therefore, be underestimated.

External validity should be noted for this meta-analysis. Included individual RCTs reported numerous exclusion criteria for patient enrollment. The results of this meta-analysis should not be generalized to patients with particular comorbidities. Most RCTs excluded patients with underlying diseases that might benefit from corticosteroids, such as inflammatory airway diseases or vasculitis. Were these patients enrolled, the study outcome might be more likely to favor the corticosteroid group. On the other hand, clinical trials also excluded patients with conditions that militate against the use of corticosteroids, such as active gastrointestinal bleeding, disseminated infections, extensive burns or immunocompromised status. Outside the scope of the generalizability of current data, the use of corticosteroids in ARDS should be individually evaluated. Underlying diseases are important considerations to justify the use of corticosteroids.

### Strengths and limitations

The strengths of our study include a comprehensive search strategy to include all studies analyzed in previous meta-analyses but not be restricted to these studies and to evaluate short-term and longer-term outcomes of corticosteroid therapy. The diversity of treatment outcomes among different etiologies of ARDS was also evaluated. These analyses help explore the causes of inconsistency among previous meta-analyses and achieve a more concrete suggestion for the use of corticosteroids in ARDS. With inclusion of the data from cohort studies, the disparity between clinical trials and real-world practice was disclosed, and their consistent results or trends helped to increase the robustness of this meta-analysis. In addition, we performed a sensitivity analysis to test the influence of study pooling strategy, an analysis that previous meta-analyses did not perform. Our study also has limitations. The number of RCTs and sample size were relatively small. There are only two studies in some subgroup analyses and underpower is a concern. Sparse data are another concern for data pooling by the random-effects model. With respect to the evaluation of an etiology-specific response to corticosteroids, the classification of ARDS etiologies was limited because the mix of study populations was diverse among several studies. Finally, study quality might be a confounding factor that we were unable to control in the subgroup analysis when we try to explore the association between follow-up duration and mortality. Earlier studies tend to be of poor quality and their follow-up duration was also shorter.

## Conclusions

ARDS is a heterogeneous disease with various etiologies and clinical courses. The effects of corticosteroids on ARDS were inconsistent in previous studies due to different outcome measures and study populations. This study shows that corticosteroids do not improve longer-term outcomes and may cause harm in certain subgroups of ARDS, such as influenza-related ARDS. Based on current available data, we do not suggest routine use of corticosteroids for ARDS. More clinical trials are needed to improve the overall quality of evidence and to specify the unfavorable and favorable subgroups of ARDS for corticosteroid therapy. Future studies should evaluate short-term as well as longer-term outcomes for comprehensive evaluation of the treatment efficacy of corticosteroids in ARDS.

## Key messages

•ARDS is a heterogeneous disease with various etiologies and clinical courses. The effects of corticosteroids on ARDS were inconsistent in previous studies due to different outcome measures and study populations.

•This meta-analysis evaluated short-term and longer-term effects of corticosteroids on ARDS mortality. Pooled data showed that corticosteroid therapy did not decrease longer-term mortality.

•The effectiveness of corticosteroid therapy differed in different etiologies of ARDS. Corticosteroids might cause harm in certain subgroups of ARDS patients, such as influenza-related ARDS.

•Current data do not support routine use of corticosteroids for ARDS. More clinical trials are needed to improve the overall quality of evidence and to specify the unfavorable and favorable subgroups of ARDS patients for corticosteroid therapy.

## Abbreviations

ARDS: acute respiratory distress syndrome; CI: confidence interval; RCT: randomized clinical trial; RD: risk difference; RR: relative risk.

## Competing interests

The authors declare that they have no competing interests.

## Authors’ contributions

SYR and HHL designed the study. SYR, HHL and CTH conducted the analysis and interpretation of the data and drafted the manuscript. PHK, HDW and CJY contributed to the interpretation of the data and critical revision of the manuscript for important intellectual content. All authors read and approved the final manuscript. SYR and HHL are guarantors.

## Authors’ information

Sheng-Yuan Ruan, MD; Graduate Institute of Epidemiology and Preventive Medicine, National Taiwan University, Taipei, Taiwan; and Division of Pulmonary and Critical Care Medicine, Department of Internal Medicine, National Taiwan University Hospital, Taipei, Taiwan; Hsien-Ho Lin, MD, ScD; Graduate Institute of Epidemiology and Preventive Medicine, National Taiwan University, Taipei, Taiwan; Chun-Ta Huang, MD, Ping-Hung Kuo, MD, Huey-Dong Wu, MD and Chong-Jen Yu, MD, PhD; Division of Pulmonary and Critical Care Medicine, Department of Internal Medicine, National Taiwan University Hospital, Taipei, Taiwan.

## Supplementary Material

Additional file 1 Contains details of included studies (e-Table S1), quality assessment of included studies (e-Table S2 and e-Table S3), comparability of included cohort studies (e-Table S4), within study comparison of different mortality endpoints (e-Table S5) and definition of infection in included studies (e-Table S6). Click here for file

Additional file 2 Contains funnel plot for outcome of mortality in randomized controlled trials (e-Figure S1) and cohort studies (e-Figure S2). Click here for file
