# Supplementary material for: Exploring the heterogeneity of effects of corticosteroids on acute respiratory distress syndrome: a systematic review and meta-analysis
Source: Crit Care. 2014 Apr 7;18(2):R63. doi: 10.1186/cc13819 (PMC4056095; doi:10.1186/cc13819)
Supplement: Additional file 1 — Contains details of included studies (e-Table S1), quality assessment of included studies (e-Table S2 and e-Table S3), comparability of included cohort studies (e-Table S4), within study comparison of different mortality endpoints (e-Table S5) and definition of infection in included studies (e-Table S6). [file cc13819-S1.pdf]

Additional file-1

Exploring the heterogeneity of effects of corticosteroids on ARDS: a systematic review and meta-analysis

Sheng-Yuan Ruan, MD; Hsien-Ho Lin, MD, ScD; Chun-Ta Huang, MD; Ping-Hung Kuo, MD; Huey-Dong Wu, MD; and Chong-Jen Yu, MD, PhD

e-Table 1. Details of included randomized controlled trials (RCTs) and cohort studies

e-Table 2. Quality assessment for randomized controlled trials using Cochrane Risk of Bias Tool

e-Table 3. Quality assessment for cohort studies using Newcastle-Ottawa Quality Assessment Scale

e-Table 4. Comparability between steroid and control groups in cohort studies

e-Table 5. Randomized trials reporting both short-term and hospital mortality

e-Table 6. Definitions of corticosteroids-associated infections in RCTs and cohort studies

e-Table 1. Details of Included Randomized Controlled Trials (RCTs) and Cohort Studies

Author (Published Year)	Timing of steroid therapy[a]	Study sites	Age[b]	Case No.		Causes of ARDS[c]	Mortality endpoint	Overall mortality
				Control	Steroid			
**RCTs**								
Weigelt et al. (1985)	Preventive	Single-center USA	46	14	25	Transfusion 36%, sepsis 32%, contusion 21%, aspiration 16%, aspiration 16%	Hospital	44%
Bone et al. (1987)	Preventive	Multicenter USA	55	38	50	Sepsis 100%	14 days	39%
Luce et al. (1988)	Preventive	Single-center USA	52	14	13	Septic shock 100%	Hospital	78%
Bernard et al. (1987)	< 3 days	Multicenter USA	55	49	50	Sepsis 27%, aspiration pneumonia 18%, pancreatitis 4%, shock 2%, fat emboli 1%, others 42%	45 days	62%
Meduri et al. (1998)	2nd week	Multicenter USA	48	8	16	Pneumonia 50%, extrapulmonary sepsis 21%, aspiration 13%, postoperative 8%, drug 8%	Hospital	63%
Annane et al. (2006)	< 3 days	Multicenter France	60	92	85	Septic shock 100%	Hospital	68%
Steinberg et al. (2006)	2nd week	Multicenter USA	49	91	89	Pneumonia 42%, sepsis 22%, aspiration 19%, trauma 14%, transfusions 1%, other 12%	60 days	29%
Meduri et al. (2007)	< 3 days	Multicenter USA	51	28	63	Pneumonia 42%, postoperative 37%, aspiration 20%, extrapulmonary sepsis 16%, Other 22%	Hospital	30%

(Continued)

e-Table 1 (Continued)

Author (Published Year)	Timing of steroid therapy[a]	Study sites	Age[b]	Case No.		Causes of ARDS[c]	Mortality endpoint	Overall mortality
				Control	Steroid			
**Cohort studies**								
Fowler et al. (1985)	No report	Multicenter USA	53	34	53	Aspiration 18%, multiple risks 16%, pneumonia 11%, bacteremia 10%, transfusion 10%, other 23%	60 days	66%
Headley et al. (1997)	3rd week	Multicenter USA	43	34	9	Pneumonia 44%, extrapulmonary infection 16%, sepsis 7%, aspiration 5%	ICU	49%
Keel et al. (1998)	3rd week	Single-center Switzerland	50	18	13	SIRS 35%, pneumonia 29%, sepsis 19%, aspiration 16%	ICU	55%
Varpula et al. (2000)	2nd week	Single-center Finland	43	15	16	Pneumonia 87%, aspiration 13%	30 days	19%
Song et al. (2003)	1st week	Single-center China	59	17	60	Pneumonia 35%, cardiopulmonary resuscitation 16%, extrapulmonary sepsis 13%, postoperative 13%, pancreatitis 12%, trauma 6%	ICU	68%
Lee et al. (2005)	< 3 days	Single-center Korea	67	8	12	Postoperative 100%	Hospital	40%
Bajwa et al. (2009)	No report	Single-center USA	63	147	30	Pneumonia 75%, septic shock 59%, sepsis 30%, aspiration 9%, multiple transfusions 8%, trauma 4%	60 days	42%

(Continued)

e-Table 1 (Continued)

Author (Published Year)	Timing of steroid therapy[a]	Study sites	Age[b]	Case No.		Causes of ARDS[c]	Mortality endpoint	Overall mortality
				Control	Steroid			
Brun-Buisson et al. (2011)	< 3 days	Multicenter France	47	125	83	H1N1 Influenza 100%	Hospital	24%
Schellongowski et al. (2011)	< 3 days	Single-center Austria	39	3	14	H1N1 Influenza 100%	ICU	41%
Linko et al. (2011)	< 3 days	Multicenter Finland	50	12	46	H1N1 Influenza 100%	Hospital	12%

SIRS =systemic inflammatory response syndrome

[a]Average duration of ARDS prior to steroids

[b]Mean or median age in years.

[c]Causes of ARDS might be multiple.

e-Table 2. Quality Assessment for Randomized Controlled Trials Using Cochrane Risk of Bias Tool

Study	Random sequence generation (Selection bias)	Allocation concealment (Selection bias)	Blinding of participants and personnel (Performance bias)	Blinding of outcome assessment (Detection bias)	Incomplete outcome data (Attrition bias)	Selective reporting (Reporting bias)	Other bias
Weigelt (1985)	Unclear risk	Low risk	Low risk	Unclear risk	Low risk	Low risk	Unclear risk
Bone (1987)	Unclear risk	Low risk	Low risk	Low risk	Low risk	Low risk	Unclear risk
Bernard (1987)	Unclear risk	Low risk	Low risk	Low risk	Low risk	Low risk	Low risk
Luce (1988)	Low risk	Low risk	Low risk	Low risk	Low risk	Low risk	Unclear risk
Meduri (1998)	Low risk	Low risk	Unclear risk	Low risk	Low risk	Low risk	Unclear risk
Annane (2006)	High Risk	Low risk	Low risk	Low risk	Low risk	Low risk	Unclear risk
Steinberg (2006)	Low risk	Low risk	Low risk	Low risk	Low risk	Low risk	Low risk
Meduri (2007)	Unclear risk	Low risk	Unclear risk	Low risk	Low risk	Low risk	Unclear risk

e-Table 3. Quality Assessment for Cohort Studies Using Newcastle-Ottawa Quality Assessment Scale

Study	Selection				Comparability	Outcome		
	Representativeness of the exposed cohort	Selection of the non-exposed cohort	Ascertainment of exposure	Demonstration that outcome of interest was not present at start of study		Assessment of outcome	Was follow-up long enough for outcomes to occur	Adequacy of follow up of cohorts
Fowler (1985)		★	★	★		★	★	★
Headley (1997)		★	★	★		★	★	★
Keel (1998)		★	★	★	★	★	★	★
Varpula (2000)		★	★	★	★	★	★	★
Song (2003)		★	★	★		★	★	★
Lee (2005)			★	★	★	★	★	★
Bajwa (2009)		★	★	★	★	★	★	★
Brun-Buisson (2011)	★	★	★	★	★ ★	★	★	★
Schellongowski (2011)		★	★	★		★	★	★
Linko (2011)		★	★	★	★	★	★	★

e-Table 4. Comparability between Steroid and Control Groups in Cohort Studies

Study	Adjusted factors or comparability statement in individual cohort study
Fowler (1985)	Nil
Headley (1997)	Comparable APACHE II score and etiology of acute lung injury between steroid and control groups.
Keel (1998)	Some attending physicians used corticosteroids for ARDS but others did not.
Varpula (2000)	Comparable distribution of age, sex, admission APACHE II score, PaO_2/FiO_2 ratio and etiology of acute lung injury between steroid and control group.
Song (2003)	Nil
Lee (2005)	Control group was identified from a period in which steroid therapy was not given for ARDS in the study hospital. The steroid and control groups were similar demographically and clinically (age, sex, operation method, onset of ARDS and PaO_2/FiO_2 ratio).
Bajwa (2009)	Using multivariate analysis to adjust for C-reactive protein level, age, APACHE III score and acute hepatic failure.
Brun-Buisson (2011)	Propensity score–adjusted Cox survival analysis.
Schellongowski (2011)	Nil
Linko (2011)	Comparable age, sex between steroid and control groups.

APACHE = Acute Physiology and Chronic Health Evaluation

e-Table 5. Randomized Trials Reporting Both Short-term and Hospital Mortality

**Study**	**Mortality endpoint**	**No. of death / No. of patients**		**Relative risk (95% C.I.)**
		**Control**	**Steroids**	
Meduri (1998)	ICU mortality	5 / 8	0 / 16	0.05 (0.003 to 0.78)
	Hospital mortality	5 / 8	2 / 16	0.20 (0.05 to 0.81)
Annane (2006)	28-day mortality	62 / 92	49 / 85	0.85 (0.68 to 1.06)
	Hospital mortality	67 / 92	54 / 85	0.87 (0.71 to 1.07)
Meduri (2007)	ICU mortality	12 / 28	13 / 63	0.48 (0.15 to 0.92)
	Hospital mortality	12 / 28	15 / 63	0.56 (0.30 to 1.03)

e-Table 6. Definitions of Corticosteroids-associated Infections in RCTs and Cohort Studies

Study	Definition of infections	Duration of evaluation	No. of patients with infection / No. of patients		Infection risk
			Control	Steroids	
**RCTs**					
Bernard (1987)	New infections with a positive culture from blood or sterile sites.	7 days	5 / 49	8 /50	10% vs 16%
Meduri (1998)	Infection surveillance including pneumonia, catheter related infection, sinusitis, urinary tract infection, bacteremia and others.	During therapy	6 / 8	12 / 16	75% vs 75%
Steinberg (2006)	Serious infections (e.g., nosocomial pneumonia, disseminated fungal infection, or sepsis).	28 days	30 / 91	20 / 89	33% vs 22%
Annane (2006)	Superinfections (Catheter-related infection, nosocomial pneumonia, urinary tract infection, surgical wound infection, others).	28 days	12 / 92	12 / 85	13% vs 14%
Meduri (2007)	Infection surveillance including pneumonia, catheter related infection, sinusitis, urinary tract infection, bacteremia and others.	7 days	8 / 28	10 / 63	29% vs 16%
		During study	17 / 28	27 / 63	61% vs 43%
**Cohort studies**					
Varpula (2000)	Nosocomial infections.	During therapy	5 / 15	9 /16	33% vs 56%
Brun-Buisson (2011)	ICU-acquired infection.	ICU stay	44 / 125	38 / 83	35% vs 46%
	ICU-acquired pneumonia.	ICU stay	33 / 125	34 / 83	26% vs 41%
