# Supplementary material for: Exploring the heterogeneity of effects of corticosteroids on acute respiratory distress syndrome: a systematic review and meta-analysis
Source: Crit Care. 2014 Apr 7;18(2):R63. doi: 10.1186/cc13819 (PMC4056095; doi:10.1186/cc13819)

Additional file-2

Exploring the heterogeneity of effects of corticosteroids on ARDS: a systematic review and meta-analysis

Sheng-Yuan Ruan, MD; Hsien-Ho Lin, MD, ScD; Chun-Ta Huang, MD; Ping-Hung Kuo, MD; Huey-Dong Wu, MD; and Chong-Jen Yu, MD, PhD

e-Figure 1. Funnel plot for outcome of mortality in randomized controlled trials. A p-value of 0.90 for Begg's test and 0.86 for Egger's test

e-Figure 2. Funnel plot for outcome of mortality in cohort studies. A p-value of 0.47 for Begg's test and 0.30 for Egger's test

e-Figure 1. Funnel plot for outcome of mortality in randomized controlled trials. A p-value of 0.90 for Begg's test and 0.86 for Egger's test.

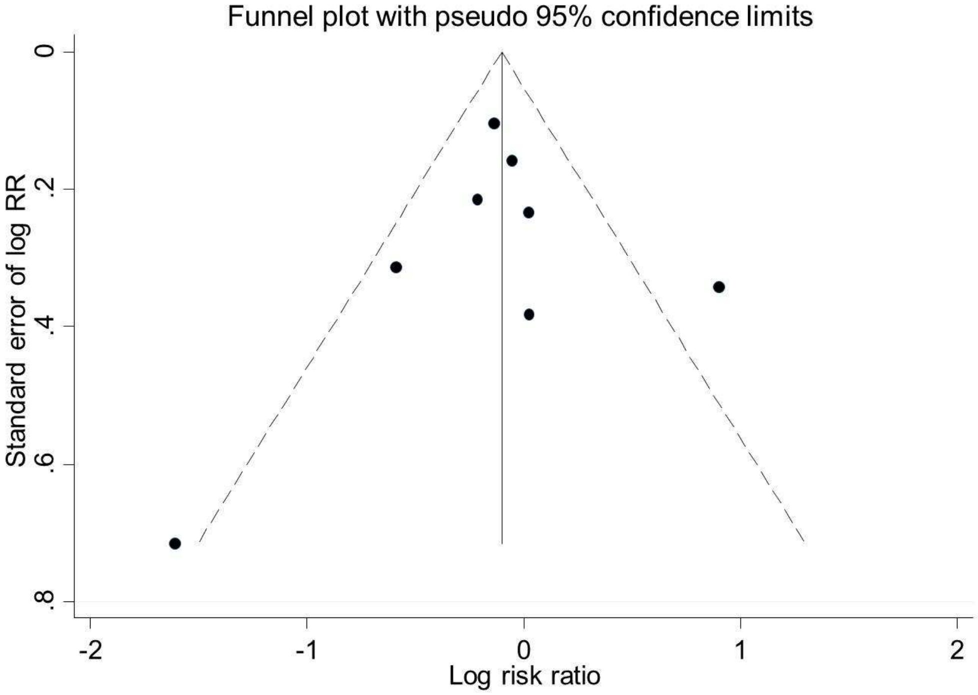

e-Figure 2. Funnel plot for outcome of mortality in cohort studies. A p-value of 0.47 for Begg's test and 0.30 for Egger's test.

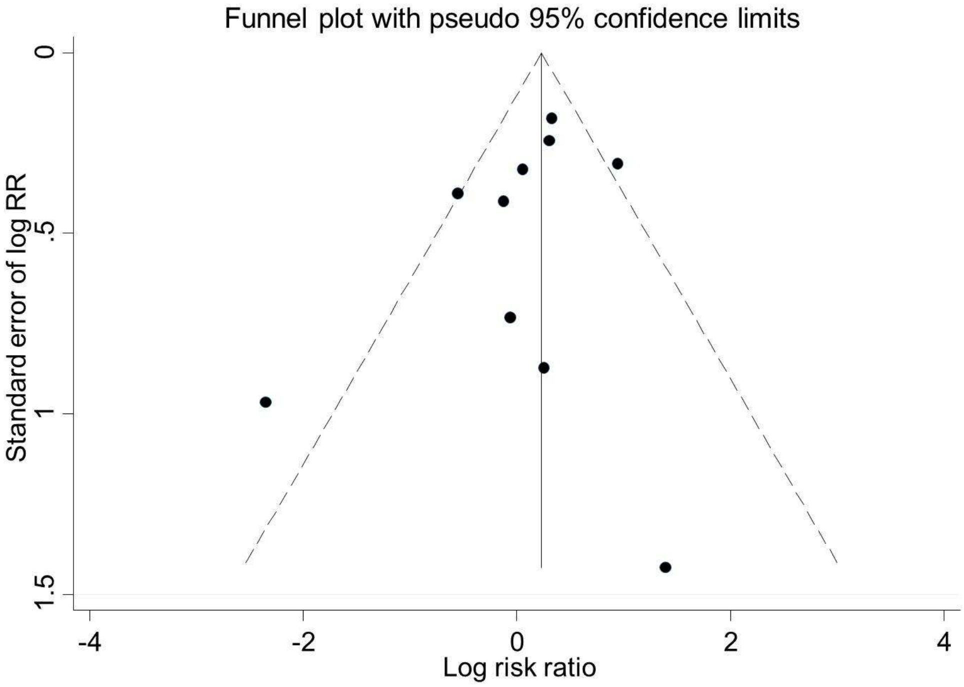

Supplement: Additional file 2 — Contains funnel plot for outcome of mortality in randomized controlled trials (e-Figure S1) and cohort studies (e-Figure S2). [file cc13819-S2.pdf]
